# Effect of an eLearning Memory Program on Reducing Negative Impact of Age-Related Memory Changes

**DOI:** 10.1093/geroni/igab046.045

**Published:** 2021-12-17

**Authors:** Lynn Zhu, Danielle D'Amico, Iris Yusupov, Jordan Lass, Brian Levine, Susan Vandermorris, Angela Troyer

**Affiliations:** 1 Baycrest, Toronto, Ontario, Canada; 2 Ryerson University, Toronto, Ontario, Canada; 3 York University, Toronto, Ontario, Canada

## Abstract

Age-related memory changes pose considerable concerns for aging adults, and can adversely affect their daily living and cause worry even when changes experienced are not clinically significant. The Memory and Aging Program® is a validated psychoeducation and memory strategy-training program that teaches the public about memory changes during aging and trains them to use evidence-based strategies to support brain health. The program has been offered in-person for over 20 years, and a self-guided eLearning version was recently developed to improve program accessibility. This study evaluated the self-reported impacts of memory changes in older adults who completed this eLearning against a control group. We randomized 202 older adults, without neurological or psychiatric diagnoses (71.6 years; 69 % female; 15.6 years of education), into the eLearning program or a control group that received no intervention. All participants reported their perceived impact of memory changes using the Memory Impact Questionnaire at pre-, post-, and 6-8 weeks follow-up. A significant reduction in negative impact of memory changes on daily living and a significant improvement in positive coping with memory changes relative to controls was observed at post-test (13.4 versus 2.5 points reduction and 7.4 versus 0.1 point improvement, respectively, both p < 0.05), but these did not persist at follow-up. The adoption of digital tools has hastened across all ages. Our study showed that self-guided digital tools, such as the eLearning Memory and Aging Program®, may be a promising avenue to help aging individuals reduce the impact of memory changes on daily living.

